# Distribution of Systemically Administered Nanoparticles Reveals a Size-Dependent Effect Immediately following Cardiac Ischaemia-Reperfusion Injury

**DOI:** 10.1038/srep25613

**Published:** 2016-05-10

**Authors:** David J. Lundy, Kun-Hung Chen, Elsie K.-W. Toh, Patrick C.-H. Hsieh

**Affiliations:** 1 Institute of Biomedical Sciences, Academia Sinica, Taipei 115, Taiwan

## Abstract

Nanoparticles represent an attractive option for systemic delivery of therapeutic compounds to the heart following myocardial infarction. However, it is well known that physicochemical properties of nanoparticles such as size, shape and surface modifications can vastly alter the distribution and uptake of injected nanoparticles. Therefore, we aimed to provide an examination of the rapid size-dependent uptake of fluorescent PEG-modified polystyrene nanoparticles administered immediately following cardiac ischaemia-reperfusion injury in mice. By assessing the biodistribution of nanoparticles with core diameters between 20 nm and 2 μm 30 minutes after their administration, we conclude that 20–200 nm diameter nanoparticles are optimal for passive targeting of the injured left ventricle.

Myocardial infarction, and the associated long-term disorder of progressive heart failure, are some of the highest causes of morbidity and mortality in the modern world. In the United States, there are close to one million myocardial infarctions per year, resulting in more deaths than cancer, respiratory disorders and accidents combined^1^.

During myocardial infarction, vast areas of cardiac tissue sustain damage and approximately one billion cells undergo necrosis and apoptosis. The wounded area is unable to sufficiently regenerate myocardium or restore functional parity due to the low proliferation rate of cardiomyocytes^2^,^3^. Instead, post-infarction fibrosis and ventricular remodelling result in an enlarged, permanently weakened cardiac tissue which is more susceptible to future failure^3^,^4^,^5^. The current best practices of treatment after myocardial infarction focus on the restoration of blood flow as soon as possible following the ischemic attack. This is typically achieved via percutaneous coronary intervention (PCI, or coronary angioplasty), or by therapeutic thrombolytic drugs^6^,^7^,^8^. These procedures allow for reperfusion and subsequent reoxygenation of ischemic tissue, minimising the degree of cardiac cell necrosis and apoptosis, as well as reducing the volume of weak, dysfunctional scar tissue which will form^8^. Combined, these interventions lower the risk of immediate death, and also reduce the rate of progression into heart failure. However, reperfusion itself bears another set of complications, with rapid pH correction, reactive oxygen species generation and a surge of calcium ions contributing towards the overall pathophysiology of myocardial infarction^9^,^10^,^11^,^12^.

Researchers have explored a broad range of therapeutic approaches, aiming to reduce cardiomyocyte death, lessen the degree of ventricular remodelling, and improve cardiac function following cardiac ischaemia. The most direct approaches typically involve the injection of cardioprotective growth factors, cytokines, antioxidants or bioactive small molecules directly into the infarcted area, or applying them in conjunction with a reparative or supportive biomaterial^13^,^14^,^15^,^16^,^17^,^18^,^19^,^20^. However, direct myocardial injection is highly invasive, volume-limited and has the potential to cause further injury to the already-weakened myocardium. Another option for delivery to the myocardium is intracoronary catheterisation, where therapeutics are delivered directly into the coronary artery. This achieves effective delivery to the injured area, and is less invasive than direct myocardial injection^21^. However, many therapeutics are not compatible with catheter-based delivery, and the procedure itself carries risk of embolisation – particularly given that the coronary arteries may be compromised following myocardial infarction^18^. Furthermore, intervention which acts directly upon the cardiac tissue soon after infarction is considered risky, due to the instability of the left ventricular wall^18^. Therefore, intravenous administration remains an attractive way to deliver therapeutics to the heart immediately following ischaemia-reperfusion injury. Intravenous administration is simple to perform, non-invasive and without the risks and costs associated with surgery or catheterisation. However, intravenous administration is limited by relatively poor delivery to the myocardial tissue compared to reticuloendothelial organs such as the liver and spleen^21^.

It has been well established that tissue vasculature becomes increasingly permeable following injury, allowing for passive targeting of therapeutics to damaged tissues. Indeed, this “enhanced permeation and retention” (EPR) effect has been exploited in the past for tumour targeting^22^,^23^. There is some previous evidence to show that the damage induced by ischaemia-reperfusion injury alters vascular permeability in the heart^24^,^25^. However, this “leaky” vasculature alone is not sufficient to ensure optimal delivery of therapeutic compounds^26^.

Many studies have shown that the physical and chemical properties of nano-carriers affect their biodistribution and retention in various tissues^27^,^28^,^29^,^30^,^31^,^32^,^33^,^34^. In particular, nanoparticle size affects sequestration by immune cells and the clearance rate from the blood stream^34^,^35^. With reference to the heart, a previous study has also shown that nano-carrier size (15 nm micelle vs 100 nm liposome) alters retention in the infarcted region following permanent coronary artery occlusion^36^. In addition, a previous study in our lab has demonstrated a size-dependent effect on nanoparticle retention by the infarcted heart, even after direct myocardial injection^20^.

Nano-carrier-based therapies remain an attractive option to deliver therapeutic molecules to target tissues since they can be administered intravenously and circulate systemically to the affected area^36^,^37^,^38^,^39^. Nano-carriers may also be used to protect sensitive molecules from degradation in circulation, thus prolonging their circulation time^40^. Nano-carriers can also be customised with targeting moieties, without modifying the therapeutic itself, and their properties may be altered to fine tune their biodistribution following systemic administration, including responding to environmental changes such as pH, temperature or reactive oxygen species^38^,^41^.

Therefore, we sought to investigate the optimal nanoparticle size for rapid passive targeting of the myocardium immediately following ischaemia-reperfusion (I/R) injury. Since rapid intervention is essential for post-myocardial infarction treatment, an optimal carrier, administered soon after injury, will be quickly taken up and retained within the injury site. Therefore, we have utilised a relatively short time interval of 30 minutes between myocardial injury and nanoparticle injection and we measured nanoparticle uptake by the heart 30 minutes following nanoparticle injection. We perfused each animal with 50 mL PBS prior to organ collection to remove free nanoparticles from circulation – thus measuring only those nanoparticles which were retained within tissues. A schematic diagram outlining this experimental procedure is shown in Fig. 1.

We have utilised an ischaemia-reperfusion (I/R) model, rather than permanent coronary artery ligation, to more accurately recapitulate the clinical situation occurring in human patients. Studies utilising simple permanent artery occlusion fail to account for reperfusion injury (including remote organ injury) which contributes towards the overall pathogenesis of myocardial infarction. Furthermore, it is expected that delivery of systemically injected compounds to the infarcted area would be compromised if the coronary artery remains occluded.

## Results

### Nanoparticle Modification and Characterisation

Prior to administration, nanoparticle core diameter was determined by transmission electron microscopy and the hydrodynamic diameter was measured by dynamic light scattering. Commercially available nanoparticles were chosen for their high degree of consistency in terms of size and shape. For all sizes of nanoparticle, the core diameter was found to be similar to the size stated by the manufacturer, as shown in Table 1. Following the attachment of polyethylene glycol (PEG), the hydrodynamic size of the nanoparticles increased and the surface charge became significantly less negative, as expected. The nanoparticle sizes used for description are referring to the stated solid core size of each nanoparticle, but the measured hydrodynamic diameter should be considered when interpreting these results, particularly for 20 nm core diameter nanoparticles which have an average hydrodynamic diameter of 65.3 nm. [Fig f1]

### High Performance Liquid Chromatography (HPLC) quantification

**Figure 2:**
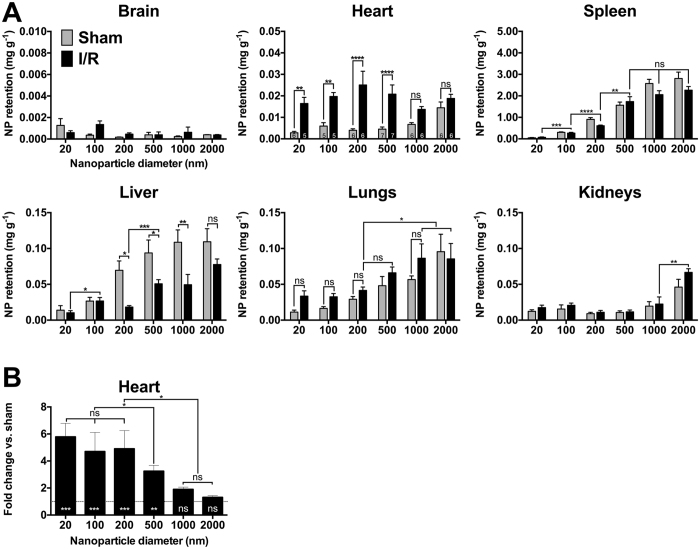
HPLC Analysis of PEG modified Fluorescent Polystyrene Nanoparticle Biodistribution Following I/R Injury. (**A**) Increased retention of nanoparticles with core diameters of 20, 100, 200 and 500 nm (hydrodynamic diameters of 65, 136, 226 and 548 nm respectively) occurs in the heart following I/R injury. Larger nanoparticles show a greater degree of off target retention, particularly by the spleen. **(B)** Expressing nanoparticle retention by the heart in terms of fold change in I/R vs. sham mice shows a clear size dependent accumulation. Statistical annotations inside the base of each bar show comparisons between retention in I/R and sham operated mice. Error bars show the standard error of the mean. The number of animals per group is shown in (**A**) for the heart. * = P ≤ 0.05, ** = P ≤ 0.01, *** = P ≤ 0.001, **** = P ≤ 0.0001, ns = P > 0.05.

**Figure 3:**
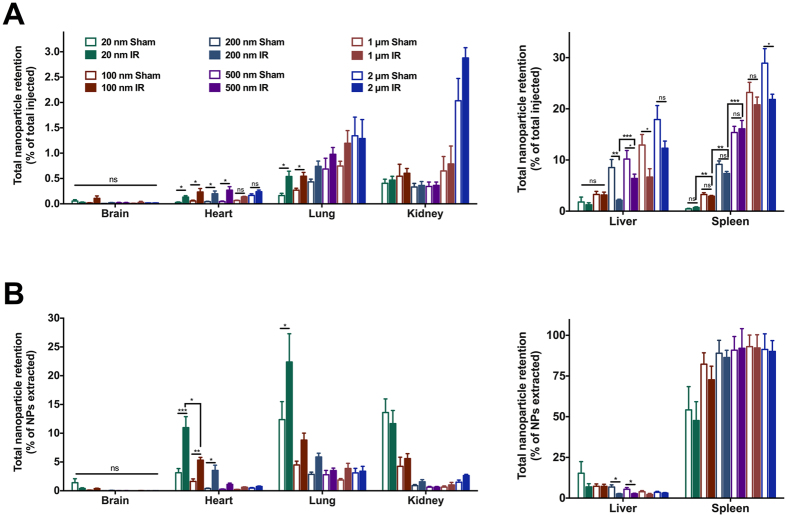
Percentage Biodistribution of PEG modified Fluorescent Polystyrene Nanoparticle Biodistribution Following I/R Injury. **(A)** Results are expressed as nanoparticle retention by each organ as a percentage of the total nanoparticle dose injected. Increased retention of 20, 100, 200 and 500 nm core diameter nanoparticles is noted in the heart following I/R injury. **(B)** Results are expressed as a percentage breakdown of nanoparticle retention measured across six vital organs, totaling 100%. * = P ≤ 0.05, ** = P ≤ 0.01, *** = P ≤ 0.001, **** = P ≤ 0.0001, ns = P > 0.05.

High Performance Liquid Chromatography (HPLC) analysis allows for highly sensitive, accurate measurement of nanoparticle retention by each organ by quantifying the amount of fluorescent dye extracted from the tissue^34^. Standard curves for each size of nanoparticle were generated by diluting nanoparticles in water across a wide concentration range, extracting the fluorescent dye and measuring the amount by HPLC. Supplemental Figure S1 demonstrates the high recovery rate of fluorescent dye from tissues. Results were then expressed as the mass of nanoparticles retained per gram of tissue (Fig. 2A), as a percentage of all injected nanoparticles (Fig. 3A) or as a percentage breakdown across the measured organs, totalling 100% (Fig. 3B).

HPLC analysis of six vital organs from sham and I/R injured mice, shown in Fig. 2A, reveals several changes in nanoparticle biodistribution following I/R injury. Uptake by the brain is very low (<0.002 mg g^−1^) for all nanoparticle sizes and does not change following I/R injury. The same trend is observed when expressing nanoparticle retention as a percentage of the total dose of injected nanoparticles (Fig. 3A) and in terms of the mass balance of all recovered nanoparticles (Fig. 3B). This is expected since the blood-brain barrier is known to prevent nanoparticles from crossing the endothelia^42^. In addition, systemic perfusion removes free nanoparticles from blood vessels. In the heart, nanoparticles with diameters of 20 nm (hydrodynamic diameter 65 nm), 100 nm (136 nm), 200 nm (226 nm) and 500 nm (548 nm) were retained in significantly greater concentrations following I/R injury than in sham controls (Fig. 2A). Nanoparticles of 1 μm and 2 μm (hydrodynamic diameters of 1099 nm and 2518 nm) showed no significant difference in retention by the heart following I/R injury. Expressing the results for the heart in terms of retention in I/R versus sham operated animals (Fig. 2B) reveals a clear size-dependent effect. PEG-modified polystyrene nanoparticles with core diameters of 20 nm, 100 nm, 200 nm and 500 nm were retained in significantly greater quantities after I/R injury than in sham-operated animals (5.7-fold, 4.7-fold, 4.9-fold and 3.9-fold higher than sham respectively). Retention of 1 μm and 2 μm nanoparticles was not significantly different to sham operated animals. There was no significant difference between the fold changes in retention of 20 nm, 100 nm and 200 nm nanoparticles. However, these three sizes were retained more than 500 nm nanoparticles, which were in turn retained more than 1 μm or 2 μm nanoparticles following I/R injury. Figure 3A confirms that a greater percentage of injected 20, 100, 200 and 500 nm nanoparticles are retained by the heart following I/R injury. However, it is clear that of all nanoparticles injected, less than 1% are retained by the heart. Expressing the results to show the biodistribution as a percentage breakdown (Fig. 3B) shows that there was a size-dependent effect. In I/R injured mice, the heart retained 11.0% of the 20 nm nanoparticles measured across all six organs, compared to 3.1% in sham operated mice. This was significantly more than the percentage of 100 nm nanoparticles (5.3%) or 200 nm nanoparticles (3.5%) retained by the heart following I/R injury.

With regard to other organs, it is notable that most injected nanoparticles are quickly retained by the spleen in accordance with their diameter, as shown in Figs 2A and 3A. Figure 3B confirms that 47.6% of the 20 nm nanoparticles retained by I/R injured mice were retained by the spleen. This percentage rises to more than 90% for nanoparticles with a core diameter larger than 200 nm. The liver is also a major site of nanoparticle retention, particularly for larger nanoparticles. Interestingly, a significant reduction in the retention of 200 nm, 500 nm and 1 μm nanoparticles by the liver was noted in I/R-injured mice, as shown in Figs 2A and 3A.

### Histological Analysis

Although HPLC is useful for quantifying the total nanoparticle retention by each organ, this technique requires destruction of the tissue and does not consider the distribution of nanoparticles within the organ itself. For delivery of cardioprotective agents, nanoparticle retention within the infarcted left ventricle is of particular interest. Therefore, we examined the hearts of sham and I/R-injured mice, using isolectin as a marker for blood vessels. Fluorescence images are best used for visualising nanoparticle location, rather than attempting to quantify their retention since the dye content, and therefore fluorescence intensity, varies between each size of nanoparticle. Furthermore, it is not possible to count individual nanoparticles due to their small size.

**Figure 4:**
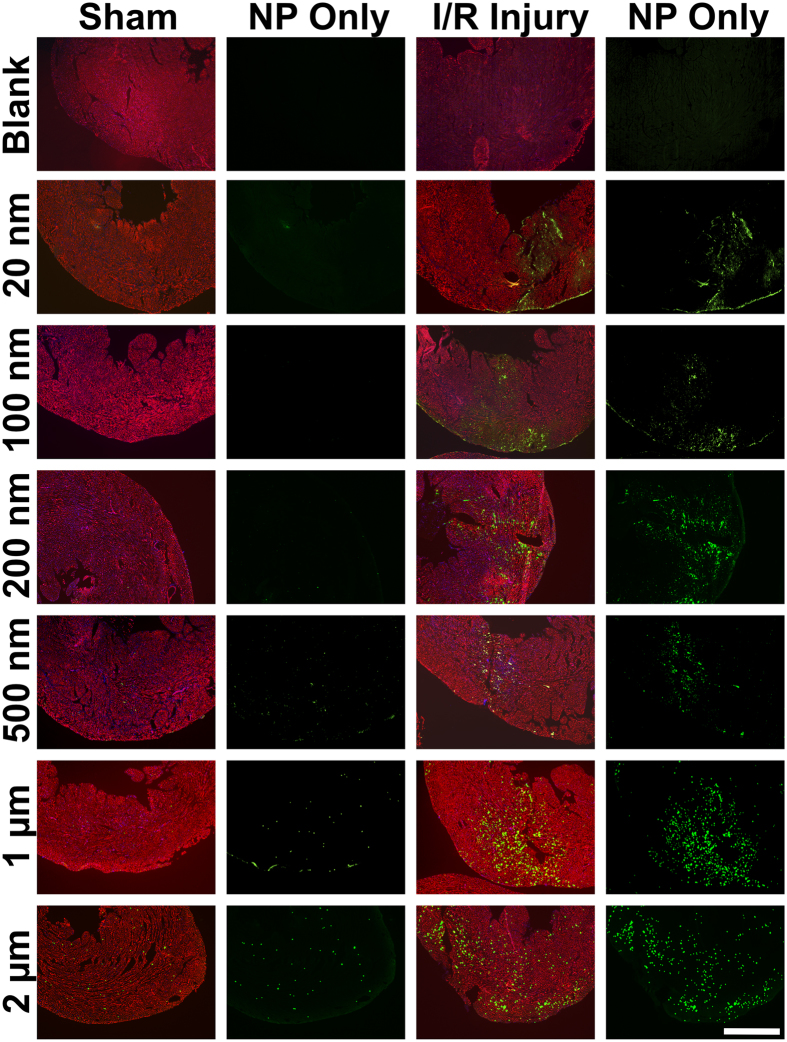
Immunofluorescence Images of the Left Ventricle showing Accumulation of Nanoparticles Following Sham Surgery or I/R Injury. Blank controls show I/R and sham hearts where no nanoparticles were administered. Frozen sections were stained for isolectin (red) and DAPI (blue). Nanoparticles are visible on the green channel. “NP only” shows the green channel alone. Scale bar = 1 mm.

**Figure 5:**
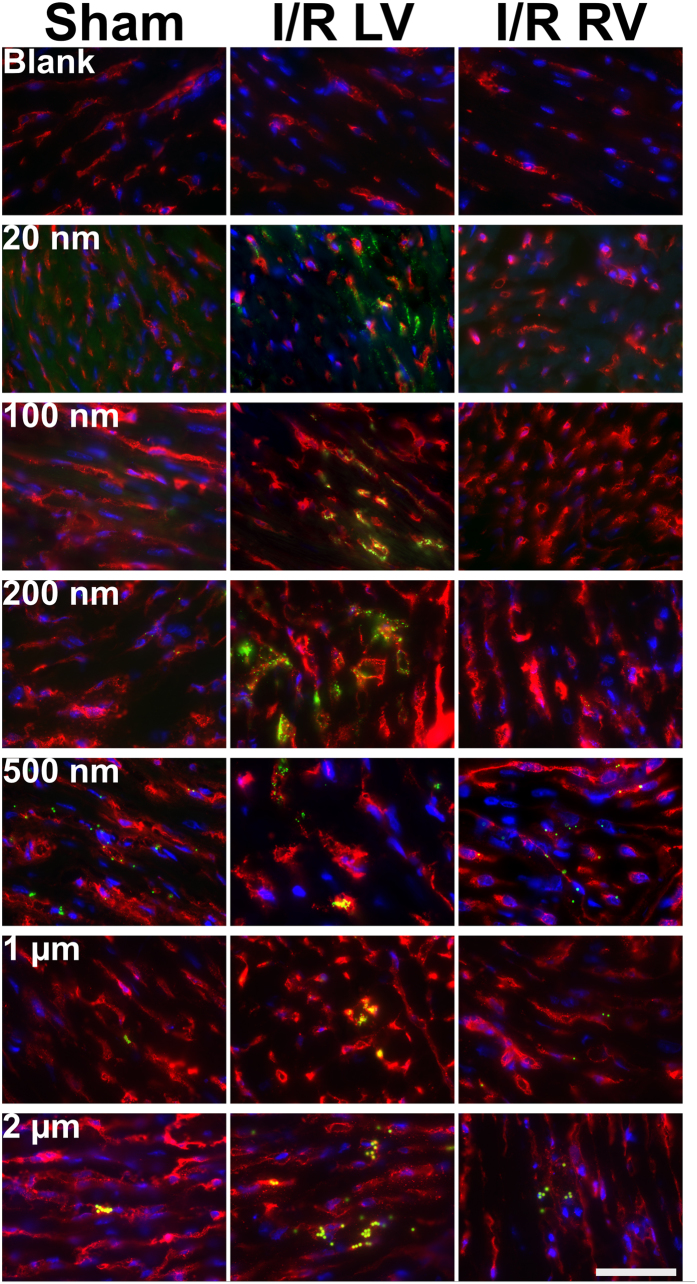
High Magnification Images Showing Nanoparticle Extravasation in the sham left ventricle, I/R injured Left Ventricle (LV) and unaffected Right Ventricle (RV). Blank controls show I/R and sham hearts where no nanoparticles were administered. Isolectin is shown in red, DAPI in blue and nanoparticles in green. Scale bar = 50 μm.

Imaging the left ventricle of sham and I/R-injured hearts (Fig. 4) shows clear differences in fluorescent nanoparticle distribution after I/R injury. Please see Supplemental Figure S2 for lower magnification images which show an entire cross section of the heart following I/R injury or sham operation. Images from blank I/R and sham controls, in which no nanoparticles were injected, demonstrate a lack of autofluorescence. Few 20 nm nanoparticles can be seen in sham-operated hearts, but a disperse cloud of nanoparticles is found in the I/R-injured left ventricle. High magnification examination of the section (Fig. 5) shows occasional 20 nm nanoparticles in sham operated hearts, mostly outside of blood vessels. Examination of the right ventricle reveals very few nanoparticles, showing that increased accumulation is mainly located in the left ventricle.

100 nm nanoparticles also appear scant in sham-operated hearts. However, after I/R injury, nanoparticle localisation in the left ventricle is clearly seen. High magnification imaging (Fig. 5) reveals almost no visible 100 nm nanoparticles in sham operated hearts, or in the right ventricle of I/R-injured hearts. However, in the I/R-injured left ventricle there is a clear accumulation of nanoparticles, visualised both inside and outside of blood vessels. On the other hand, 200 nm nanoparticles can be observed sparsely distributed throughout the sham-operated heart. High magnification analysis shows that these nanoparticles are predominantly inside blood vessels. In I/R-injured hearts there is an accumulation of nanoparticles at the left ventricle, with nanoparticles present both inside and outside of blood vessels. Occasional 200 nm nanoparticles can also be observed in the uninjured right ventricle of I/R-injured hearts (Fig. 5). 500 nm nanoparticles can be observed distributed throughout the entire left and right ventricles of sham-operated hearts. In I/R-injured hearts there is colocalisation with the injured area, and high magnification examination, combined with isolectin staining, reveals that the majority of 500 nm nanoparticles are located within blood vessels. 1 μm particles are found throughout the entire heart tissue in sham operated animals, and the right ventricle of I/R-injured hearts. Nevertheless, following I/R, there still appears to colocalisation with the left ventricle. High magnification imaging shows that these nanoparticles are invariably contained within blood vessels. 2 μm particles are also found throughout the entire heart tissue of both sham and I/R-injured animals and also appear to show some relocation to the left ventricle. High magnification images reveal large clusters of these particles, seemingly entrapped within narrow blood vessels.

## Discussion

We have found that a wide size range of nanoparticles will co-localise with the heart soon after I/R injury. However, we conclude that nanoparticles with a core diameter in the 20–200 nm range are optimal for rapid passive targeting of the I/R-injured left ventricle. Both 20 nm and 100 nm nanoparticles showed very low retention in sham operated hearts, but significantly increased retention following I/R injury (5.7-fold and 4.7-fold respectively). Immunofluorescence imaging also confirmed that 20 nm and 100 nm nanoparticles were present mostly outside of blood vessels in the infarcted left ventricle. 200 nm nanoparticles also showed a significant increase in retention following I/R injury (4.9-fold). However, immunofluorescence staining revealed that many 200 nm nanoparticles were contained inside blood vessels of the heart, and HPLC analysis showed significantly higher concentrations of 200 nm nanoparticles were retained by the spleen than 100 nm (2.3-fold) or 20 nm (8.7-fold) nanoparticles. 500 nm nanoparticles also showed significantly increased retention in the heart following I/R injury. However, nanoparticles of 500 nm and larger showed much greater off-target retention in the spleen.

It is apparent that immunofluorescence imaging of 1 μm and 2 μm nanoparticles appears to show effective passive targeting of the left ventricle. However, HPLC-based quantification of nanoparticle retention confirms the increased retention of 1 μm nanoparticles is not quite statistically significant (p = 0.08), and 2 μm nanoparticles are not significantly different after I/R injury (p = 0.24). Careful examination of histology sections reveals that 1–2 μm nanoparticles were distributed throughout the entire heart, even in healthy animals. This agrees with HPLC analysis which shows a higher retention of larger nanoparticles in sham-operated mice (0.015 mg g^−1^ for 2 μm, compared to 0.002–0.004 mg g^−1^ for 20–500 nm nanoparticles). Interestingly, these larger nanoparticles within the infarct zone appeared as clusters, mainly inside blood vessels (Fig. 5). Since these nanoparticles are PEG-modified, they should not aggregate, and systemic perfusion would remove freely circulating nanoparticles. Therefore, we presume that these larger micro-sized particles may be physically entrapped within small capillaries of the heart. These clusters of nanoparticles have an extremely high fluorescence intensity, which we believe contributes towards the appearance of increased accumulation, particularly in low magnification images. In any case, the high degree of accumulation in the lungs, kidneys, liver and spleen limit the attractiveness of these larger particles for targeting the infarcted myocardium. These results clearly reinforce the need for accurate quantification by methods such as HPLC during biodistribution studies, rather than relying on immunofluorescence imaging alone to estimate nanoparticle retention.

Interestingly, a significant reduction of nanoparticle retention by the liver was noted for some sizes of nanoparticles following I/R injury. While we did not directly investigate the underlying mechanisms behind this altered distribution, there is published evidence showing that the liver is prone to remote organ injury due to reactive oxygen species generated upon coronary artery reperfusion^43^. The liver has also been shown to respond to cardiac ischaemia-reperfusion injury by releasing a variety of pro-inflammatory cytokines and growth factors, as well as mobilising cells, which may play a role in cardioprotection^44^. Assessing the percentage distribution of nanoparticles (Fig. 3B) shows a reduction in nanoparticle retention by the liver in terms of mass balance compared to other organs. Therefore, we speculate that a combination of hemodynamic changes and local changes to vascular permeability in the liver, may have led to the altered distribution noted in our study.

We remind the reader that our conclusions are likely to be material dependent to some extent and that differences in the composition of other nano-carriers may produce different results. Nevertheless, these results highlight the importance of correct size selection for passive nano-carrier targeting. Indeed, the fold change difference we measured between differently sized nanoparticles is larger than the change measured with some actively targeted methods^37^,^45^,^46^. Another factor to consider when interpreting these results is the short time duration between nanoparticle administration and organ collection. We aimed to determine which nanoparticle size could be retained by the infarction area immediately following reperfusion, since this is an ideal therapeutic window for cardioprotection^9^. Therefore these results do not capture the ultimate fate of injected nanoparticles, which may occur over a longer time period – particularly for the non-degradable polystyrene nanoparticles used in our study^47^. Finally, these results serve as a reminder that passive nanoparticle uptake by the heart following injury remains very low. Regardless of which nanoparticle size is utilised, a large proportion of systemically nanoparticles are retained by reticuloendothelial organs within 30 minutes.

## Methods

### Material sources

FluoSphere Carboxylate-Modified Yellow-Green Fluorescent Microspheres (core diameters of 0.02, 0.1, 0.2, 0.5, 1.0 and 2.0 μm) were purchased from Life Technologies, Thermo Fisher. Product numbers were F8787, F8803, F8811, F8813, F8823, F8827 respectively. Methoxypolyoxyethylene amine (mPEG-amine), MW = 5,000 was obtained from Nanocs, Taiwan. N-Hydroxysulfosuccinimide (Sulfo-NHS) was obtained from Thermo Scientific. N-(3-Dimethylaminopropyl)-N′-ethylcarbodiimide hydrochloride (Carbodiimide) was obtained from Sigma-Aldrich. All organic solvents were of HPLC grade and all aqueous solutions were prepared with deionised water.

### Animal Experimentation

Animals used in this experiment were 8 week old, male, FVB mice purchased from Biolasco, Taiwan. Mice were kept in a 12 hour day/night cycle with ad libitum access to food and water. All experimentation was approved by the Experimental Animal Committee, Academia Sinica, Taiwan, and carried out in accordance with their guidelines. For HPLC analysis, the number of animals used per group was as follows; Ischaemia-reperfusion: 20 nm, n = 5; 100 nm, n = 5; 200 nm, n = 6; 500 nm, n = 7; 1 μm, n = 6; 2 μm, n = 6. Sham: 20 nm, n = 6; 100 nm, n = 5; 200 nm, n = 6; 500 nm, n = 7; 1 μm, n = 6; 2 μm, n = 6. For histology analysis, a minimum of a further two animals per group were analysed, and representative images are shown. Animal surgery was performed by an experienced lab technician who has performed over 500 rodent myocardial infarction and ischaemia-reperfusion operations. All operations were carried out by the same technician. 8 week old male FVB mice were anesthetised with isofluorane/air, ventilated and monitored throughout the entire procedure. Ischaemia was induced via reversible ligation of the left anterior descending coronary artery. After 45 minutes of ischaemia, reperfusion was allowed for 30 minutes. Sham operations were performed as a control. Following surgery, mice were randomly assigned to nanoparticle administration groups. Nanoparticles were administered *via* lateral tail vein at a dose of 40.0 μg nanoparticles per g body weight.

### PEGylation reaction

For PEGylation of nanoparticles ≥100 nm, 60 mg Methoxypolyoxyethylene amine (mPEG-amine, MW = 5,000) was dissolved into 500 μl polystyrene nanoparticle solution. 500 μl 0.2 M borate buffer (pH 8.2) was added and the pH was adjusted to 7.8 using 0.5 N NaOH. 14 mg Sulfo-NHS was added, mixed for 5 minutes, and 100 μl carbodiimide (EDC) (20 mg/ml in 50 mM MES buffer, pH 6.0) was added. The resulting solution was then mixed for 6 hours in the dark. Free mPEG-amine was removed using Amicon ultracentrifuge filters (MWCO 30/50 KDa) and the PEGylated nanoparticle solution was resuspended with deionised water pH 7.0 and quantified by HPLC. For 20 nm polystyrene nanoparticles, the procedure was modified slightly. Nanoparticles were first concentrated by ultracentrifugation and resuspended with 1 ml 50 mM MES buffer pH 6.0. 90 mg methoxy-PEG5000-NH2 was added and the solution was stirred for 15 minutes followed by the procedure as described above.

### Characterisation of Nanoparticles

Stock and PEG-modified polystyrene nanoparticles were characterised by hydrodynamic diameter and zeta potential using a Malvern Zetasizer Nano ZS. PEGylated nanoparticles were diluted in deionised water for hydrodynamic sizing or diluted in 10 mM TrisHCl pH 7.0 for zeta potential measurement. For TEM analysis, nanoparticles were deposited onto a copper grid and stained with 1–2% v/v phosphotungstic acid solution, then dried overnight. The average core diameter was calculated by measuring ≥100 nanoparticles in ImageJ.

### Nanoparticle dye extraction and quantification

Mice were subjected to systemic perfusion with 50 ml heparinised saline via the abdominal aorta. Organs were collected, cut into several pieces for ease of homogenisation, weighed, and 500 μl deionised water was added. Samples were thoroughly homogenised with zirconia beads in 30 second bursts for a total of 5 minutes using a Roche MagNA Lyser instrument. 500 μl *o*-xylene was added, and samples were sonicated in 10 second bursts for a total of 2 minutes with vigorous mixing between each round. Samples were frozen at −80 °C for 30 minutes, allowed to thaw at room temperature, then spun at 14,000 rpm for 30 minutes. Supernatant, containing the fluorescent dye extracted from lysed nanoparticles, was then analysed by HPLC. HPLC quantification was performed using a Waters e2695 separation module. The mobile phase was 77:23 methanol:water (1 mL min^−1^ flow rate) and separation was achieved using an X-bridge C18 (250 × 4.6 mm, 5 μm) column at 40 °C. Detection was performed using a Waters 2475 FLR Detector (excitation 505 nm, emission 515 nm). Unknown quantities of fluorescent dye extracted from tissues were quantified using a standard curve created from known concentrations of nanoparticles diluted in water with their fluorescent dye extracted. The fluorescent dye recovery rate was assessed by spiking known concentrations of nanoparticle solution into organs from mice who had not received nanoparticle injection, and extracting fluorescent dye as described previously. We have previously demonstrated the recovery rate of fluorescent dye to be ≥90% across the full range of concentrations described^34^. A further evaluation of the recovery rate is shown in Supplemental Figure S1.

### Immunofluorescence staining

Following systemic perfusion, the heart was quickly removed, washed, fixed, cryopreserved, embedded in OCT and sectioned in 7 μm sections. Slides were washed with PBS and blocked (5% goat serum, 5% FBS) then anti-isolectin antibody conjugated to AlexaFluor 647 (ThermoFisher) was applied at room temperature for 1 hour. Slides were then washed, counterstained with DAPI and mounted. All images were taken using a Zeiss AxioScop A1 and AxioCam MRm and Zeiss AxioVision software. Isolectin staining was visualised on the far-red channel (Cy5 filter, 590–650 nm), nanoparticles on the green channel (FITC filter, 465–495 nm) and DAPI on the blue channel (DAPI filter 325–375 nm).

### Software and Statistical Handling

Data are presented as mean ± standard error of the mean. Statistical significance and the number of samples is noted in figure legends where appropriate. Figures were assembled in Apple Keynote and Affinity Designer (Mac). Images in Supplemental Figure S2 were assembled in Microsoft Composite Image Editor. Brightness adjustments were made to immunofluorescence images to allow clearer visualisation of nanoparticle locations within tissue sections. Changes in nanoparticle biodistribution between sham and I/R-injured mice for each nanoparticle size (HPLC, Figs 2A and 3A,B) were analysed by 2-way ANOVA (Tukey’s multiple comparison test). Differences in nanoparticle retention in sham vs. I/R hearts (Fig. 2B) and recovery rate of nanoparticle dye from tissues (Supplemental Figure S1) were assessed by t-test.

## Additional Information

**How to cite this article**: Lundy, D. J. *et al.* Distribution of Systemically Administered Nanoparticles Reveals a Size-Dependent Effect Immediately following Cardiac Ischaemia-Reperfusion Injury. *Sci. Rep.* **6**, 25613; doi: 10.1038/srep25613 (2016).

## Supplementary Material

Supplementary Information

## Figures and Tables

**Figure 1 f1:**
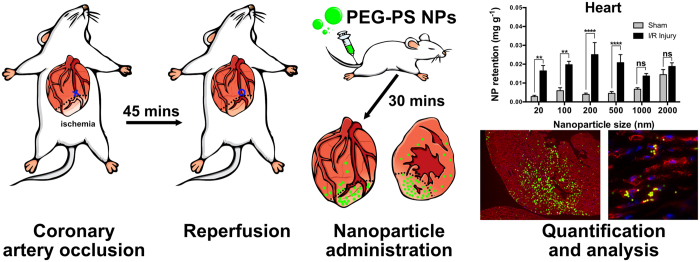
Schematic Overview of Experimental Procedures. Mice were subjected to 45 minutes of cardiac ischemia, followed by reperfusion for 30 minutes. PEG modified polystyrene nanoparticles (core diameters 20 nm–2 μm) were then injected by tail vein and allowed to circulate for 30 minutes. Animals were perfused with 50 ml heparinized saline before organs, including the heart, were collected. Nanoparticle retention was then quantified by HPLC and the locations of nanoparticles within the heart were visualized by immunofluorescence imaging.

**Table 1:** Nanoparticle Characterization.

Nanoparticle	Core Diameter, TEM (nm ± SD)	Hydrodynamic Size, DLS (nm)	Zeta potential (mV)
20 nm PS	22.1 ± 2.7	32.4 ± 0.2	−31.8 ± 0.5
20 nm PEG-PS		65.3 ± 0.3	−3.2 ± 1.1
100 nm PS	96.6 ± 6.2	121.8 ± 0.5	−37.0 ± 1.5
100 nm PEG-PS		136.4 ± 1.5	−3.9 ± 0.6
200 nm PS	188.1 ± 7.2	212.3 ± 1.1	−55.2 ± 2.4
200 nm PEG-PS		225.8 ± 1.7	−3.7 ± 0.9
500 nm PS	454.1 ± 15.8	500.7 ± 4.4	−42.7 ± 1.3
500 nm PEG-PS		548.1 ± 7.3	−3.4 ± 0.2
1 μm PS	1010.6 ± 42.0	1028.1 ± 75.1	−51.0 ± 1.3
1 μm PEG-PS		1099.0 ± 21.7	−4.4 ± 0.1
2 μm PS	1970.0 ± 93.8	2339.0 ± 52.1	−52.1 ± 3.7
2 μm PEG-PS		2518.3 ± 121.9	−4.3 ± 0.2

Core diameter was measured by TEM, and hydrodynamic size was measured by DLS. Hydrodynamic size increased compared to the core diameter, and zeta potential became less negative following PEG modification, as expected.
