# Supplementary material for: Distribution of Systemically Administered Nanoparticles Reveals a Size-Dependent Effect Immediately following Cardiac Ischaemia-Reperfusion Injury
Source: Sci Rep. 2016 May 10;6:25613. doi: 10.1038/srep25613 (PMC4861966; doi:10.1038/srep25613)
Supplement: Supplementary Information [file srep25613-s1.pdf]

Supplemental Information

SREP-16-10931

Distribution of Systemically Administered Nanoparticles Reveals a Size-Dependent Effect Immediately following Cardiac Ischaemia-Reperfusion Injury

David J. Lundy[1], Kun-Hung Chen[1], Elsie K.-W. Toh[1], and Patrick C.-H. Hsieh[1*]

[1] Institute of Biomedical Sciences, Academia Sinica, Taipei 115, Taiwan
[*] Corresponding author phsieh@ibms.sinica.edu.tw

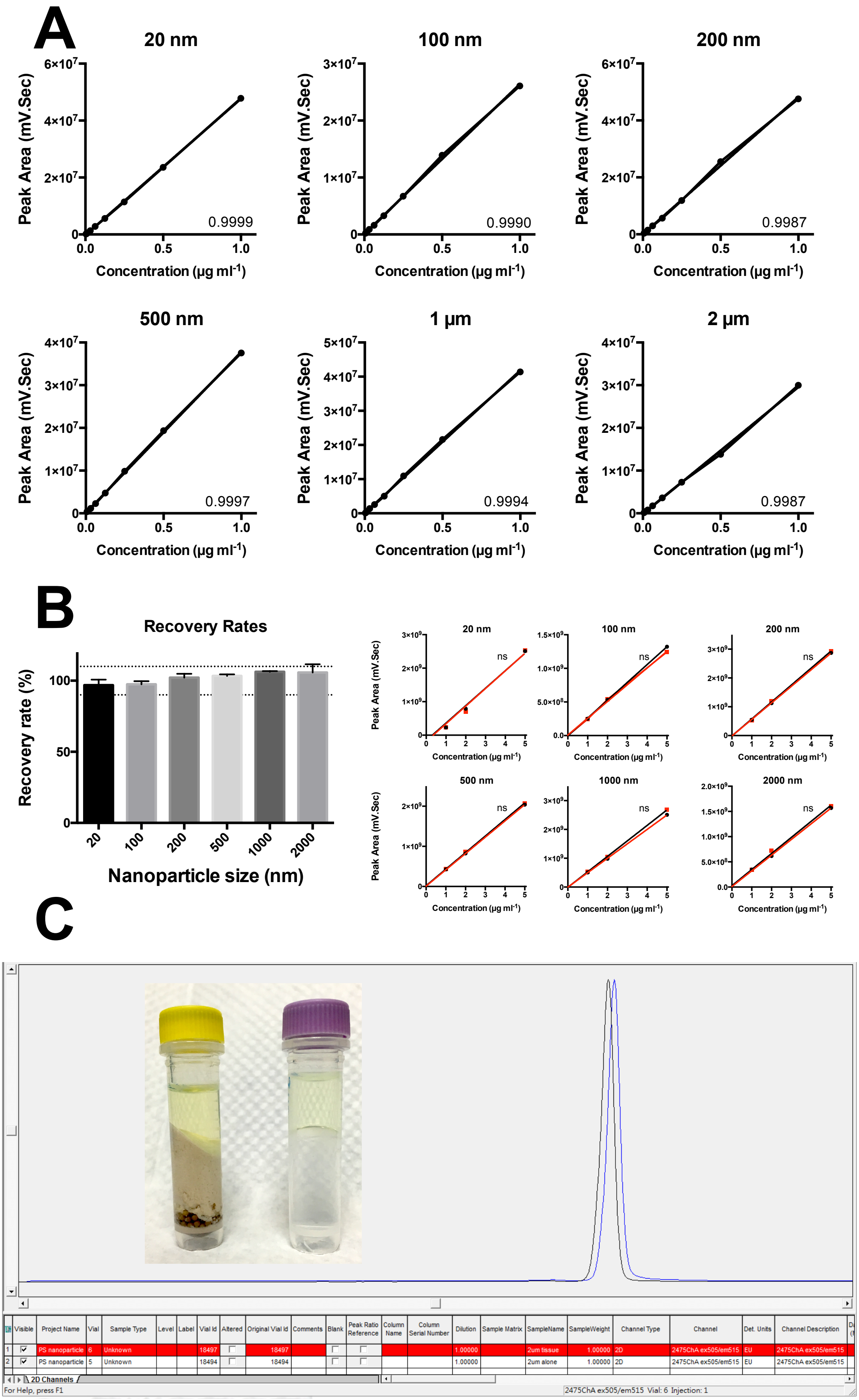
A
20 nm
100 nm
200 nm
500 nm
1 μm
2 μm
Peak Area (mV.Sec)
Concentration (μg ml-1)
0.9999
0.9990
0.9987
0.9997
0.9994
0.9987
B
Recovery Rates
Recovery rate (%)
Nanoparticle size (nm)
20
100
200
500
1000
2000
ns
C
Visible | Project Name | Vial | Sample Type | Level | Label | Vial Id | Altered | Original Vial Id | Comments | Blank | Peak Ratio Reference | Column Name | Column Serial Number | Dilution | Sample Matrix | SampleName | SampleWeight | Channel Type | Channel | Det. Units | Channel Description
1 PS nanoparticle 6 Unknown 18497 18497 1.00000 2um tissue 1.00000 2D 2475ChA ex505/em515 EU 2475ChA ex505/em515
2 PS nanoparticle 5 Unknown 18494 18494 1.00000 2um alone 1.00000 2D 2475ChA ex505/em515 EU 2475ChA ex505/em515
2D Channels
For Help, press F1
2475ChA ex505/em515 Vial: 6 Injection: 1

Supplemental Figure 1. Validation of HPLC-based quantification methodology. (A) Linear standard curves for nanoparticles of each diameter. R-squared values are shown. (B) The recovery rate of fluorescent dye from nanoparticles spiked into tissue samples, compared to the dye extracted from nanoparticles alone. For all nanoparticle sizes, the recovery rate is approximately 100%. Dotted lines denote 90% and 110%. $n \geq 3$ for each measurement. Error bars show the standard error of the mean. Standard curves showing dye extracted from nanoparticles alone (black lines) and the same concentration of nanoparticles spiked into tissue samples (red line). The GraphPad Prism linear regression "are lines different" tool was used to compare each standard curve. For all nanoparticle sizes, the standard curves are considered to be equal. (C) Sample HPLC peaks for dye extracted from 2 µm nanoparticles alone (black line) or the same concentration of 2 µm nanoparticles spiked into tissue samples (blue line). The peak volumes for the two measurements were 347529793 and 340724952 respectively (< 2 % difference). Inset, photo showing fluorescent dye extracted from 250 mg tissue (left) and from nanoparticles alone (right), used for this HPLC measurement.

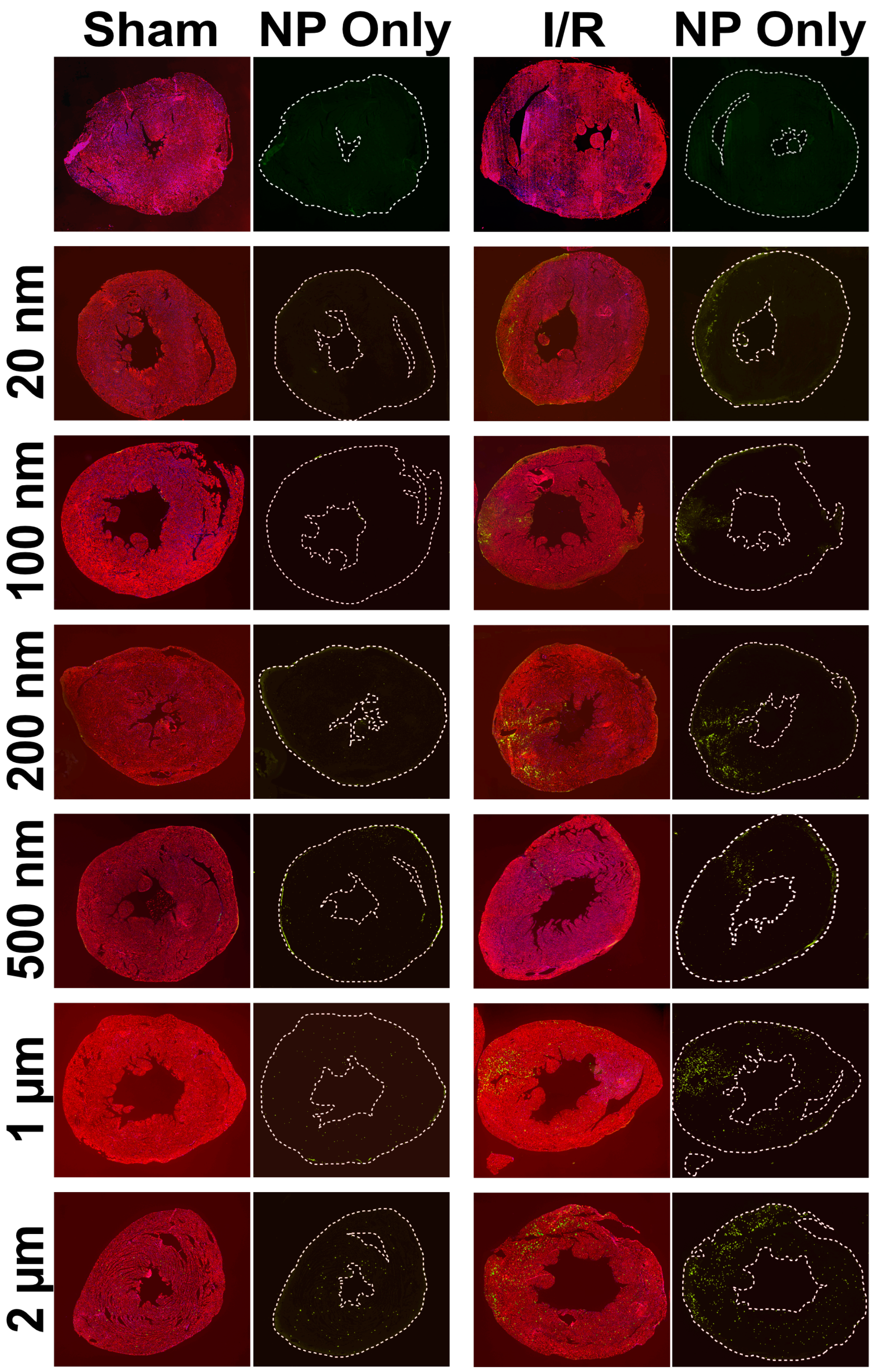

Supplemental Figure 2. Low Magnification Images of Sham and I/R-Injured Hearts. NP only shows the green channel alone.
